# Spinal Anæsthesia

**Published:** 1921-01-15

**Authors:** Henry Robinson


**The Editor's Letter Box—(*continued*).**

### Spinal Anæsthesia.

*To the Editor of* The Hospital.

Sir,—The able advocacy of spinal anæsthesia in your issue of January 1, 1921, p. 309, will undoubtedly do good by calling attention to the precision and safety with which this method (still distrusted by too many surgeons and practitioners) can and should be employed. At the same time there are, I think, a few emendations or additions necessary in the article as published: some of these I propose to offer, in no carping spirit, please believe.

The expression "essential factor" in the second sentence of the article is unfortunate, because the presence of glucose (thus denominated) in the anæsthetic solution, so far from being essential, is even of dubious value altogether. For some years I worked with glucose solutions as described originally, I think, by Professor Barker and lauded in your article; but five or six years ago I was persuaded to try a solution of stovaine in saline, which I found infinitely preferable in every way. I know that many administrators hold the same view, and I regret that your article hardly mentions this method, and recommends the use of either dextrin or dextrose vehicles. One out of several advantages possessed by the saline solution is the possibility of using the Trendelenburg posture. Your article does not warn administrators that this is dangerous when a dextrose solution is used; possibly the author does not believe that this danger exists, but the belief in it is fairly widely spread amongst anæsthetists.

Again, Jonnesco's method is mentioned in some detail. It is, certainly, damned with faint praise; but the uninitiated would hardly infer from the wording of the paragraph what the actual truth is, namely, that the method was unanimously condemned twelve years ago as very dangerous and unwarrantable in any circumstances whatever.

From the absence of remarks on antiseptic precautions I infer that the author of your article is so modern that he does not realise how recent is the pre-antiseptic period. Still, he ought to make it clear to the weaker brethren—of whom even in the newest medical generation there are some to be found—that the strictest asepsis is *de rigueur* in the technique of spinal anæsthesia. He ought, I think, also to have mentioned that sterilising the trocars, cannulas, syringes, etc., by boiling in water containing alkali will result in the precipitation of stovaine from its solution when the syringe is filled. My own practice, for what it is worth, is to sterilise the apparatus by immersion in spirit for twenty minutes; the spirit is got rid of before usage by drawing sterile distilled water through the syringe and cannula. Another useful refinement of technique not mentioned in the article is the use of an inner cannula of fine calibre for the actual injection; this minimises wastage of stovaine solution left in the cannula when the piston of the syringe has been pressed home, and therefore conduces to more accurate dosage. When this inner cannula is used, the cerebro-spinal fluid will no longer make its way into the syringe unaided; to carry out this manœuvre the piston of the syringe must be actually withdrawn gently by the administrator.—I am, Sir, yours faithfully,

Henry Robinson, M.D.

